# RANK–RANKL signalling in cancer

**DOI:** 10.1042/BSR20160150

**Published:** 2016-08-05

**Authors:** Nathalie Renema, Benjamin Navet, Marie-Françoise Heymann, Frédéric Lezot, Dominique Heymann

**Affiliations:** * INSERM, UMR 957, Equipe Labellisée Ligue 2012, Physiopathologie de la Résorption Osseuse et Thérapie des Tumeurs Osseuses Primitives, Université de Nantes, 1 Rue Gaston Veil, 44035 Nantes, France; † Nantes University Hospital, Nantes 44035, France; ‡ Department of Oncology and Human Metabolism, The University of Sheffield, Sheffield S10 2RX, U.K.

**Keywords:** microenvironment, oncogenesis, RANK, RANKL

## Abstract

Oncogenic events combined with a favourable environment are the two main factors in the oncological process. The tumour microenvironment is composed of a complex, interconnected network of protagonists, including soluble factors such as cytokines, extracellular matrix components, interacting with fibroblasts, endothelial cells, immune cells and various specific cell types depending on the location of the cancer cells (e.g. pulmonary epithelium, osteoblasts). This diversity defines specific “niches” (e.g. vascular, immune, bone niches) involved in tumour growth and the metastatic process. These actors communicate together by direct intercellular communications and/or in an autocrine/paracrine/endocrine manner involving cytokines and growth factors. Among these glycoproteins, RANKL (receptor activator nuclear factor-κB ligand) and its receptor RANK (receptor activator nuclear factor), members of the TNF and TNFR superfamilies, have stimulated the interest of the scientific community. RANK is frequently expressed by cancer cells in contrast with RANKL which is frequently detected in the tumour microenvironment and together they participate in every step in cancer development. Their activities are markedly regulated by osteoprotegerin (OPG, a soluble decoy receptor) and its ligands, and by LGR4, a membrane receptor able to bind RANKL. The aim of the present review is to provide an overview of the functional implication of the RANK/RANKL system in cancer development, and to underline the most recent clinical studies.

## INTRODUCTION

In a physiological context, a healthy tissue microenvironment provides an adapted 3D microarchitecture with essential intercellular signalling, thus ensuring appropriate function. This tissue homoeostasis acts as a barrier to tumour development by inhibiting excessive cell growth and/or migration. Indeed, this fragile equilibrium can be destabilized by any alterations to cell communications, or interaction between cells and extracellular matrix components and consequently can become a fertile environment for cancer cells, promoting their malignant transformation and their proliferation [1]. The conjunction between one or more oncogenic events and this fertile environment can lead to the development of a tumour mass, which is frequently linked to the tumour cells escaping from the immune system [2]. In fact, this description reflects the “seed and soil” theory proposed by Stephan Paget in 1889 to explain preferential metastatic sites depending on tumour subtype [3].

This “soil” or tumour microenvironment is a very complex and dynamic organization, defined by three main “niches” depending on their functional implication: (i) an immune niche involved in local immune tolerance, (ii) a vascular niche associated with tumour cell extravasation/migration and (iii) a metastatic niche (e.g. bone, lung, liver) hosting the metastastic tumour cells [4,5]. The notion of tumour niche was initially described for haematopoietic stem cells, for which the bone microenvironment is composed of complex signalling pathways that carefully regulate stem cell renewal, differentiation and quiescence [6]. The concept of tumour niche was then extended to bone metastases, such as breast or prostate cancers [7–9]. Lu et al. [10] described a model of bone metastasis dormancy in breast cancer where VCAM-1, aberrantly expressed, promoted the transition from indolent micrometastasis to proliferating tumour by recruiting and activating *in situ* osteoclastic cells. More recently, Wang et al. [11] analysed the distribution of human prostate cancer cell lines colonizing mouse bones after intracardiac injection of tumour cells and demonstrated that homing of prostate cancer cells was associated with the presence of activated osteoblast lineage cells. These two recent manuscripts are perfect examples of the involvement of the tumour environment in the biology of bone metastases.

The tumour microenvironment thus provides all the factors necessary for cancer cell survival, dormancy, proliferation or/and migration [10] and very often, tumour cells divert this environment in their favour [7–9]. Indeed, this specific microenvironment has recently been involved in the maintenance of cancer cell dormancy [12–14] and may also play a part in drug resistance mechanisms by controlling the balance between cell proliferation and cell death, or by secreting soluble factors that dysregulate the cell cycle checkpoints, the cell death associated signalling pathways, or drug efflux [15,16].

Cell communications in physiological and pathological conditions are promoted by physical contacts involving adhesion molecules and channels, but also by a very high number of soluble mediators called cytokines and growth factors which appear to be the key protagonists in the dialogue established between cancer cells and their microenvironment [16]. These polypeptidic mediators perform their activities in an autocrine, paracrine or juxtacrine manner leading to inflammatory foci and the establishment of a vicious cycle between cancer cells and their local niches [17–19]. These proteins also have endocrine activities and contribute in this way to both the formation of a chemoattractant gradient and the metastatic process.

Considerable diversity in the cytokines and growth factors playing a role in cancer development has been identified in the last four decades. Some of them can be considered to be biological markers for aggressiveness, or to be prognostic factors, whereas others are also regarded as therapeutic targets. Among cytokine families, in the last 15 years, the biology of receptor activator nuclear factor-κB ligand (RANKL) and its receptor RANK has been widely studied in cancer [20–23] and has been identified as a key therapeutic target in numerous cancer entities, as described below. The present review gives a synthesis of RANK/RANKL pathway involvement in the carcinogenesis process. Their direct or indirect activities in oncogenic events will be described, as will their recent therapeutic applications.

## RANKL/RANK SYSTEM: DISCOVERY, MOLECULAR AND FUNCTIONAL CHARACTERIZATION

**Figure 1:**
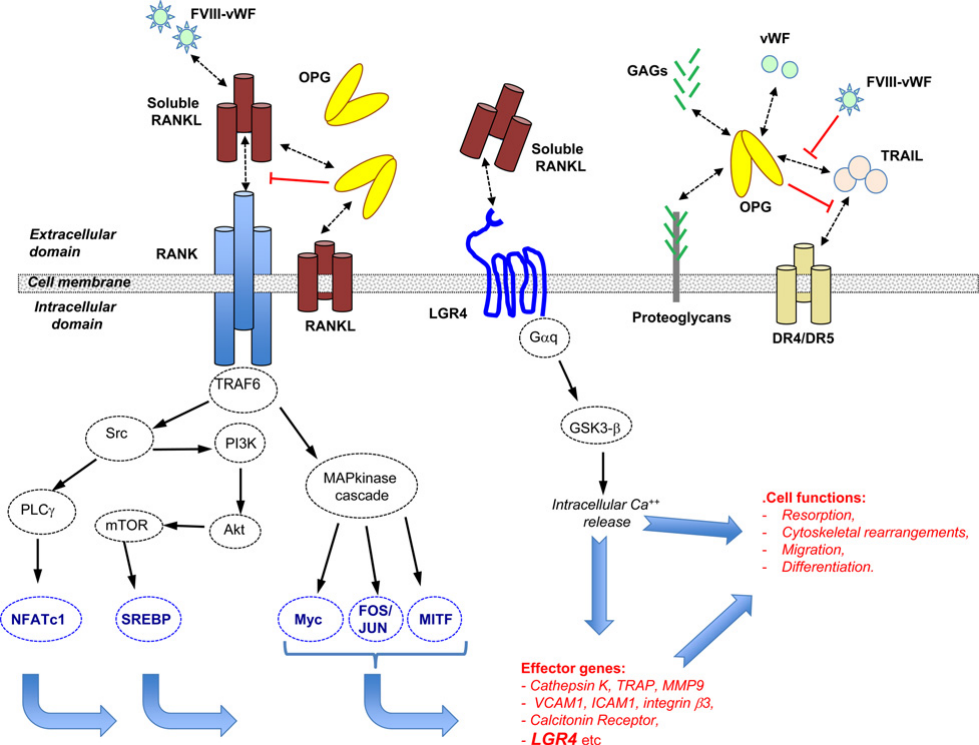
RANK/RANKL signalling in cancer cells: a very complex molecular network RANKL is a trimeric complex produced in a membrane or soluble form. Secreted RANKL can be produced from a specific transcript or by proteolysis of its membrane form. Trimeric RANKL interacts with a trimeric receptor named RANK and triggers a signalling cascade controlling the transcription of numerous effector genes. Additional protagonists intervene to regulate the binding of RANKL to RANK. In this way, OPG acts as a decoy receptor interacting with RANKL, and complex VIII (FVIII-vWF) showed a similar capacity. However, OPG is itself controlled by many ligands, including TRAIL, vWF and glycoaminoglycans (GAGs), and the final inhibitory effect of OPG on RANKL is dependent on its binding to these ligands. Very recently, it has been demonstrated that LGR4 is a new receptor for RANKL which can counterbalance the RANKL activities transmitted by RANK signalling.

The superfamily of tumour necrosis factor-α (TNFα) is composed of more than 40 members and is associated with a similar number of membrane or soluble receptors. RANKL is one member of the TNF-α superfamily (TNFSF11) and binds to a membrane receptor named receptor activator of nuclear factor-κB (RANK), a member of the TNF receptor superfamily (TNFRSF11A) [20–30]. The interactions between RANKL and RANK lead to specific intracellular signal transduction and are controlled by a decoy receptor called osteoprotegerin (OPG) (TNFRSF11B) [27] (Figure 1).

### RANKL

RANKL has alternatively been called tumour necrosis factor-related activation-induced cytokine (TRANCE) [26], osteoprotegerin ligand (OPGL) [27,28] and osteoclastic differentiation factor (ODF) [29,30]. Although RANKL is the name commonly used, the official nomenclature of this cytokine is TNFSF11. RANKL is a homotrimeric type II membrane protein with no signal peptide and existing in three isoforms due to alternative splicing of the same gene [31]. Among these isoforms, the full-length RANKL is called RANKL1, RANKL2 is a shorter form of RANKL1 in which a part of the intra-cytoplasmic domain is missing and RANKL 3 is a soluble form of RANKL, with the N-terminal part of the amino acids deleted [31]. A soluble RANKL can also result from the sheding of membrane-RANKL induced by various enzymes such as the metalloproteinase disintegrin TNF-α converting enzyme (TACE) [32] or ADAM-10, MMP-7, MMP-14 [33,34]. RANKL is expressed by a wide variety of tissues such as the brain, skin, intestine, skeletal muscle, kidney, liver, lung and mammary tissue, but is more highly expressed in bone tissue [35], lymphoid organs and the vascular system [36]. The control of bone remodelling is the predominant function of RANKL. Indeed, RANKL effectively regulates the bone resorption process by stimulating osteoclast differentiation and osteoclast survival [37,38]. Whether RANKL is expressed by osteoblasts, osteocytes, chondrocytes or stromal cells, osteocytes are its main source in adult bone [39,40]. The role of RANKL is not restricted to the bone tissue and RANKL also plays an important role in the immune system, increasing the ability of dendritic cells to stimulate both naive T-cell proliferation and the survival of RANK^+^ T-cells [25,26,41]. In this context, Wong et al. [27] demonstrated that RANKL is a specific survival factor for dendritic cells. Overall, RANKL is one of the key factors at the crossroad between bones and immunity, a topic called “osteoimmunology” [42].

### RANK

RANK, also known as TRANCE receptor [43] and TNFRSF11A, is the signalling receptor for RANKL [25]. RANK belongs to the TNF superfamily receptors and is a type I transmembrane protein. This receptor has a large cytoplasmic domain at its C-terminal domain, a N-terminal extracellular domain with four cystein-rich repeat motifs and two N-glycosylation sites [21]. Its last domain is involved in the interaction with RANKL and the induction of the receptor's trimerization [44,45]. RANK mRNAs have been detected in many tissues such as the thymus, mammary glands, liver and prostate, but more significantly in bone [21,25]. By transducing the cell signalling initiated by RANKL, RANK plays a part in controlling bone remodelling and immunity [46,47]. Its functional activities have been clearly established by studying the phenotype of RANK knockout mice which exhibit severe osteopetrosis, with a lack of mature osteoclasts, and an absence of lymph node development with impairment in B- and T-cell maturation [48,49]. RANK is then the second key protagonist of “osteoimmunology” [50].

## RANK/RANKL AND CANCER

### RANK expression identifies cancer cells as RANKL targets

The expression of RANK/RANKL is not restricted to healthy tissues and numerous studies have demonstrated their expression in neoplastic tissues. This wide distribution strengthens the hypothesis of their key role in the oncogenic process (Table 1). Thus, a high percentage of carcinoma cells express RANK mRNA/protein at various levels [51,52]. Indeed, 89% of all the carcinomas assessed exhibit RANK positive immunostaining, and approximately 60% of cases showed more than 50% of positive cancer cells. Interestingly, RANK expression in carcinoma cells is a poor prognostic marker as demonstrated in breast cancer [86,87]. Similarly to prostate cancers, Pfitzner et al. [87] demonstrated that higher RANK expression in the primary breast tumour was associated with higher sensitivity to chemotherapy, but also a higher risk of relapse and death despite this higher sensitivity. RANK expression was also described as being predictive of poor prognosis in bone metastatic patients but not in patients with visceral metastases [88]. Similarly, sarcoma cells also express RANK (18–69% depending on the series) [79,89,90] and expression is correlated with clinical parameters. Trieb and Windhager [89] described a reverse correlation between RANK expression and the overall survival of patients with osteosarcoma, but not with the response to chemotherapy. These authors observed lower disease-free and overall survival rates in patients presenting RANK positive tumours. Bago-Horvath et al. revealed that RANKL expression was significantly more common in osteosarcoma of the lower extremity than in any other location and did not find any significant correlation between RANKL and disease-free or osteosarcoma-specific survival. However, they did report that RANK expression is a negative prognostic factor regarding disease-free survival, confirming the data obtained by Trieb and Windehager [89]. Interestingly, in 2012, Papanastasiou et al. [91] identified a new isoform of RANK (named RANK-c) generated by alternative splicing and expressed in breast cancer samples. Its expression was reversely correlated with histological grade and RANK-c was able to inhibit cell motility and the migration of breast cancer cells by interfering with RANK signalling.

**Table 1:** RANK and RANKL expression in cancers

Cancer subtypes or related organ	RANK expressing tumours (references)	RANKL expressing tumours (references)
Bladder carcinoma	[51]	–
Breast carcinoma	[51–56]	[57–60]
Cervical cancer	[51,61]	[61]
Chondrosarcoma	[62]	[62,63]
Colon and rectal cancers	[51]	–
Endometrial tumours	[51]	–
Oesophageal tumours	[51,64]	–
Giant cell tumours of bone	[65]	[63–66]
Hepatocarcinoma	[51,67]	[67,68]
Lung cancer	[51,69]	[69]
Lymphoma	[51,70]	[71,72]
Melanoma	[73,74]	–
Myeloma	[75]	[75,76]
Neuroblastoma	[51]	[77]
Oral squamous carcinoma	[78]	[78]
Osteosarcoma	[63,79]	[63,79,80]
Prostate carcinoma	[51,73,81,82]	[81,83]
Renal carcinoma	[84]	[84]
Thymic tumours	[51]	–
Thyroid adenocarcinoma	[51,85]	[85]

In several studies [87,90], RANKL expression was not correlated with any clinical outcomes in either carcinoma or sarcoma. However, in one series of 40 patients, Lee et al. [92] showed that RANKL expression was related to poor response to preoperative chemotherapy and a high RANKL level was associated with inferior survival. Recently, Cathomas et al. [93] described an interesting clinical case of an osteosarcoma patient treated with sorafenib and denosumab. RANK and RANKL were expressed by the tumour cells and the authors observed complete metabolic remission for over 18 months strengthening the potential therapeutic value of blocking RANK/RANKL signalling in osteosarcoma [93]. Whereas RANK is expressed by various cancer cell types, its ligand can be produced either by tumour cells or by their environment (Table 1). Consequently, RANKL can then act in a paracrine or autocrine manner on cancer cells. The best example of such paracrine activity is given by the role of RANK/RANKL in the pathogenesis of giant cell tumours in bone. RANK is expressed by giant osteoclasts and the macrophagic component of the tumours, whereas RANKL is produced by stromal cells. Furthermore, exacerbated production of RANKL by stromal cells is directly associated with an increase in osteoclastogenesis and bone destruction [94]. This observation identifies the giant cell tumours in bone as very good candidates for the clinical use of Denosumab [95].

### Direct RANK/RANKL signalling in cancer cells: the regulatory activities of OPG and LGR4

RANK, like the other receptors in the TNF receptor superfamily, is characterized by the absence of tyrosine kinase activity and consequently requires adapter proteins named TNF-receptor associated factor (TRAF) in order to transmit cell signalling. The intracellular domain of RANK has two TRAF binding sites able to interact with TRAF-2, -3, -5 and -6 [96,97], but only TRAF6 mutations led to an osteopetrotic phenotype similar to the phenotype of RANK knockout mice, thus underlining the predominant role of TRAF6 in RANK associated signalling among the TRAF family members [96–101]. Consecutively, TRAF6 leads to the activation of Src/PLCγ, PI3K/Akt/mTOR and MAPK (p38, JNK, ERK1/2) cascades which result in the translocation of transcriptional activators including NF-κB, Fos/Jun or MITF and subsequently to the transcription of numerous effector genes involved in bone resorption such as cathepsin K or TRAP, in cell adhesion and motility such as VCAM1 or ICAM1. This explains the various functional impacts that RANKL has on normal and cancer cells (Figure 1).

The first identified regulator of RANKL activities was a soluble protein named OPG [102,103]. OPG is considered to be a ubiquitous protein with predominant expression in bone (osteoblasts, mesenchymal stem cells), immune cells (dendritic cells, T- and B-cells) and vessels (endothelial and vascular smooth muscle cells) [21,104]. OPG acts as a decoy receptor for RANKL, and blocks the RANK–RANKL interaction and RANKL-induced signalling pathways with its N-terminal [11,89]. OPG and RANKL expression are both regulated by inflammatory cytokines released into the microenvironment of cancer cells, and RANKL activities will result from the level of expression and the kinetics of both factors in this microenvironment [21,105]. OPG binds to soluble and membrane RANKL and strongly controls RANKL bioavailability at the cell membrane by facilitating its internalization and reducing its half-life [106]. However, OPG possesses numerous other ligands which markedly regulate its expression and have an impact on RANKL availability (Figure 1) [104]. In this way, OPG binds to glycosaminoglycans and proteoglycans such as syndecan-1 through its heparin-binding domain with a strong influence on cancer cell development [104,107]. The best illustration of the functional consequence of this interaction in cancer is given by myeloma cells which overexpress syndecan-1 [108]. OPG produced in the bone microenvironment is trapped, internalized and degraded by myeloma cells and the OPG/RANKL balance is then dysregulated in favour of RANKL. The OPG/RANKL imbalance leads to bone resorption, a phenomenon exacerbated by the RANKL production of the myeloma cells. By sequestering OPG, myeloma cells elaborate a microenvironment that facilitates their expansion. Similarly, OPG can be trapped by the proteoglycans and glycosaminoglycans located in the extracellular matrix as shown in osteosarcoma [109]. In addition, OPG binds TRAIL (TNF related apoptosis inducing ligand), a key natural pro-apoptotic and “anti-cancer” factor [110]. By this way, OPG can thus act as an anti-apoptotic and a pro-proliferative factor for cancer cells by blocking TRAIL activity, as shown with prostate carcinoma for instance [111]. Complex VIII (factor VIII-von Willebrand factor) is also able to bind to OPG and increases the complexity of this system by regulating TRAIL-induced cancer cell death [112]. Finally, RANKL expressed by the tumour cells or/and their environment by exerting its action through RANK in an autocrine, endocrine or paracrine manner contributes to establishing the fertile soil needed for tumour cells to be maintained and proliferate. In this picture, OPG and its ligands are notably involved in the bioavailability and biological activities of RANKL.

Very recently, a new RANKL receptor named leucine-rich repeat-containing G-protein-coupled receptor 4 (LRG4) characterized by seven transmembrane regions, has been identified [113]. In this work, Luo et al. [113] revealed that RANKL binds to the extracellular domain of LGR4 and by this way negatively regulates osteoclastogenesis through activation of Gαq/GS3K-β signalling and repression of the NFATc1 pathway (Figure 1). Moreover, *Lgr4* is a transcriptional target of the canonical RANKL–NFATc1, which shows that LGR4 signalling acts as the feedback loop controlling RANKL activities. Interestingly, a mutation in LGR4 encoding gene has been related to an osteoporosis phenotype which can be explained by the new function of LGR4 as a RANKL receptor [114]. Although the involvement of the LRG4–RANKL axis in cancer has not yet been clearly determined, LGR4 nevertheless promotes the proliferation of various tumour cells, including breast, prostate, gastric and hepatic cancer [115]. This proliferation effect was linked to activation of the Wnt/β catenin signalling pathways. LRG4 appears to be a new regulator for prostate development and promotes tumorigenesis [116,117] and the LRG4-Stat3 molecular pathway may control osteosarcoma development [118].

**Figure 2:**
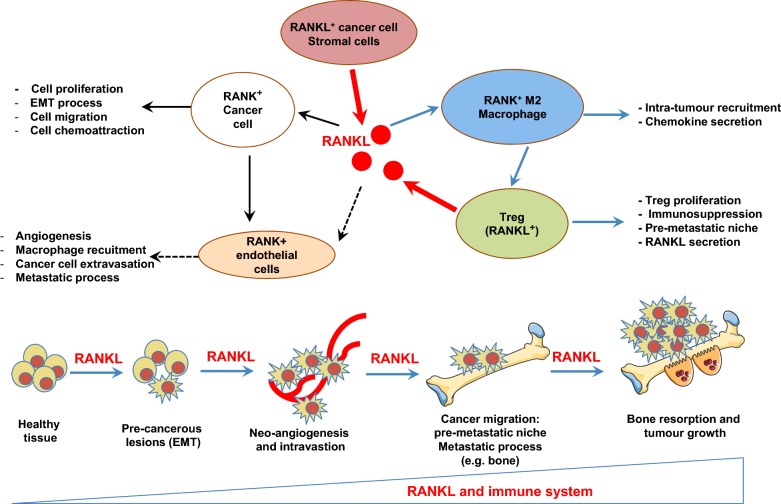
RANK/RANKL is involved in each stage of cancer development: from pre-cancerous lesions to the establishment of metastases Cancer cells are direct targets for RANKL. RANKL initiates the formation of pre-cancerous lesions by facilitating the EMT process and stemness, as well as facilitating tumour growth and the metastatic process by modulating immune and vascular niches. Throughout these processes, RANKL acts as a chemoattractive factor for cancer cells and M2 macrophages. Activated macrophages facilitate both the proliferation of Treg lymphocytes, the main source of RANKL during primary tumour growth, and the initiation of the pre-metastatic niche in bone. RANKL up-regulates the angiogenic process by stimulating the proliferation and survival of endothelial cells and, in parallel, of the metastatic process by promoting the extravasation/intravasation of RANK-expressing cancer cells and their migration to distant organs. The RANKL concentration gradient drives the tumour cells to the metastatic sites.

RANKL activities are modulated by the balance between RANKL and their various molecular regulators produced in the microenvironment of cancer cells. RANKL is involved in each stage of tumour development, from the initial oncogenesis process to the establishment of the distant metastases as described below (Figure 2).

### The RANK/RANKL axis is involved in the initial phases of tumour development

Initially considered to be a pro-metastatic factor, our vision of RANKL changed when the factor was linked to mammary gland development [119]. RANKL deficiency leads to a defect in the formation of the lobo-alveolar structures required for lactation [120,121]. In addition, RANKL is able to promote the survival and proliferation of epithelial cells simultaneously with the up-regulated expression of RANK during mammary gland development [119–121]. Disturbance in this coordinated mechanism can lead to the formation of pre-neoplasias and subsequently to that of tumour foci, as revealed by Gonzalez-Suarez et al. [122]. These authors established a mouse mammary tumour virus–RANK transgenic mice overexpressing the protein in mammary glands–and reported a high incidence of pre-neoplasia foci (multifocal ductal hyperplasias, multifocal and focally extensive mammary intraepithelial neoplasias), as well as the development of adenocarcinoma lesions in these transgenic mice compared with the wild-type mice. Confirming the involvement of RANKL in the initial oncogenic process, administration of RANK-Fc decreased both mammary tumorigenesis and the development of lung metastases in MMTV-*neu* transgenic mice, a spontaneous mammary tumour model [122]. In a complementary work, this team demonstrated that the RANKL/RANK axis was pro-active in epithelial mesenchymal transition (EMT), promoted cell migration simultaneously with neo-vascularization, and that their expression was significantly associated with metastatic tumours [123]. Overall, their data revealed that RANK/RANKL signalling promotes the initial stage in breast cancer development by inducing stemness and EMT in mammary epithelial cells. A similar process has been confirmed in head and neck squamous carcinoma [124], and in endometrial cancer [125], and RANKL expression has been associated with the EMT and appears to be a new marker for EMT in prostate cancer cells [83].

### RANK/RANKL system controls cell motility and consequently contributes to the metastastic process concomitantly with a pro-angiogenic function

Jones et al. [95] provided the first evidence of a chemoattractant activity for RANKL. These authors demonstrated that RANKL produced by osteoblasts and bone marrow stromal cells attracts RANK-expressing cancer cells and induces their migration. This mechanism seems to be relatively universal and was observed in prostate cancer [95,126,127], breast cancer [95], colon cancer [58], melanoma [95], oral squamous carcinomas [128], lung cancer [129], hepatocarcinoma [130], endometrial cancer [131], osteosarcoma [132,133] and renal cancer [134]. RANKL-induced migration is associated with specific signalling cascades, especially the activation of MAP Kinase pathways. The RANKL/RANK axis then regulates cancer cell migration and RANKL acts as a chemoattractive agent on cells that express one of their receptors.

In addition to its direct effects on cancer cells, RANKL is notably able to modulate the tumour microenvironment, in particular the formation of new blood vessels. Blood vessels are used by cancer cells to deliver large quantities of nutriments and are their main means of migrating so as to invade distant organs. RANK expression was detected in endothelial cells, and by interacting with this receptor, RANKL impacts the angiogenic process by both stimulating angiogenesis through an Src and phospholipase C-dependent mechanism [135,136], and increasing cell survival in a PI3k/Akt-dependent manner [137]. RANKL also induced the proliferation of endothelial cell precursors and the neoformation of vascular tubes [138]. This phenomenon is exacerbated by VEGF, which is frequently secreted by cancer cells and which up-regulates the RANKL response of endothelial cells by an up-regulation of RANK expression and an increase in vascular permeability [139]. These works strengthen the role of RANK/RANKL axis plays in the metastatic process by regulating cancer cell migration and the neoangiogenesis.

### Immune cell regulation by RANK/RANKL: setting up fertile soil for cancer cells

RANKL influences the microenvironment of cancer cells by acting on local immunity. The major role of RANKL in the immune system was initially identified in RANKL-knockout mice in which the development of secondary lymphoid organs was impaired, especially the lymph nodes [140,141], but also at the “central” level, where the maturation of the thymic epithelial cells necessary for T-cell development was affected [142,143]. RANKL is also involved in modulating the immune response by inducing T-cell proliferation [25] and dendritic cell survival [26]. T-cells activated as a result of RANKL expression stimulate dendritic cells, expressing RANK, to enhance their survival and thereby increase the T-cell memory response [25]. More recently, Khan et al. [144] demonstrated that RANKL blockade can rescue melanoma-specific T-cells from thymic deletion, and increases the anti-tumour immune response as shown in melanoma.

Tumour-associated macrophages (TAMs) accumulate in the tumour microenvironment and, depending on their M2 or M1 phenotype, play a part in tumour growth, angiogenesis and metastasis [145]. RANK is present at the cell membrane of monocytes/macrophages and RANKL acts as a chemoattractant factor for these cells [146]. The M2-macrophages which mainly express RANK is strongly associated with the angiogenic process [147]. RANK/RANKL signalling in the M2-macrophages modulates the production of chemokines, promoting the proliferation of Treg lymphocytes in favour of an immunosuppressive environment [148]. In breast carcinoma, RANKL is mainly produced by Treg lymphocytes (CD4^+^CD25^+^ T-lymphocytes expressing Foxp3). In this context, a vicious cycle is established between TAMs, Treg and tumour cells resulting in tumour growth, the spread of cancer cells and amplification of the metastatic process [149]. In fact, T-lymphocytes appear to be the principal source of RANKL in tumorigenesis. Whether RANKL-producing T-lymphocytes are involved in the initial step of metastatic process or not, T-lymphocytes induce a permissive environment initiating the pre-metastastic niche [150].

### RANK/RANKL and bone niche: ongoing clinical trials

When proliferative tumour cells are located in the bone environment (primary bone tumours or bone metastases), they dysregulate the balance between bone apposition and bone resorption in order to create a favourable microenvironment for their growth [151]. In this way, this bone microenvironment becomes a source of therapeutic targets, RANKL being one of them [152]. OPG-Fc was the first generation of drug targeting RANKL to be assessed in potsmenopausal women [152]. Nevertheless, due to its ability to bind to multiple ligands, and particularly to TRAIL, OPG-Fc based clinical trials have been suspended until the development of a monoclonal antibody targeting RANKL [153]. Denosumab, a fully-humanized antibody targeting RANKL and blocking its binding to RANK, has been developed to bypass this risk [51]. In osteoporotic patients, Denosumab was well-tolerated and a single s.c. dose resulted in a prolonged decrease in bone turnover [154]. The value of blocking RANKL activities has been also demonstrated by the inhibition bone resorption in numerous pre-clinical models of primary bone tumours (Ewing sarcoma [155], osteosarcoma [156,157]), bone metastases (breast [158], prostate [159], non-small cell lung cancer [160]) and in myeloma [161]) and in numerous phase II and III clinical trials (Table 2). In breast and prostate carcinoma patients, bone turnover markers were reduced in a way similar to that in the osteoporosis context and, in addition, delayed the onset of the first skeletal-related event and the risk of multiple SRE [162]. A comparison with bisphosphonate therapy demonstrated the superiority of Denosumab concerning the two previous parameters even if the overall survival rate was similar with both drugs. Additional clinical trials in metastatic diseases are currently in progress and their results will be very informative with regard to the clinical extension of Denosumab in oncology.

**Table 2:**
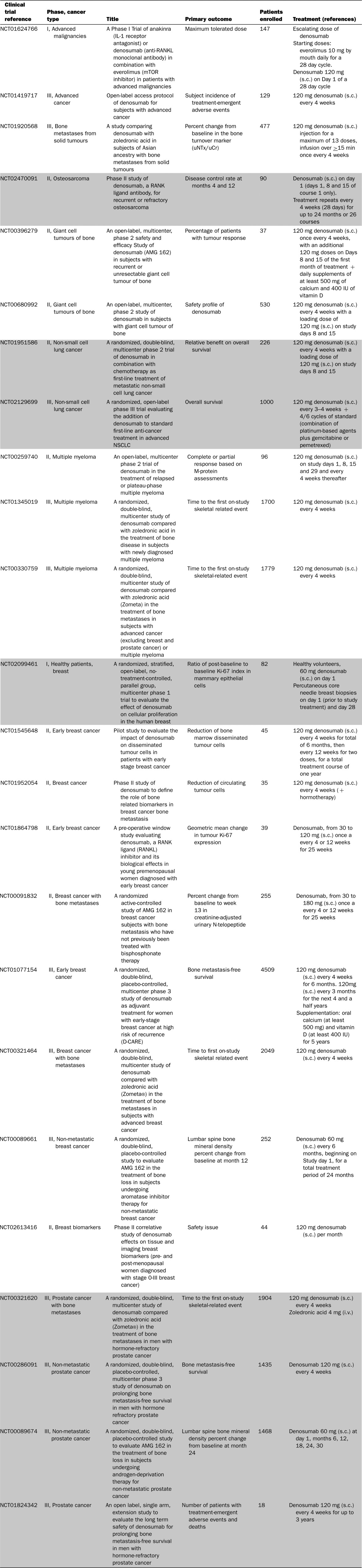
Main clinical trials based on RANKL targeting in cancers *Source*: clinical trial.gov March 2016.

## CONCLUSIONS

Since their initial discovery in 1997, RANK/RANKL became key actors in first bone remodelling and then more recently in oncology. This molecular axis is clearly involved in all stages of tumorigenesis, including tumour hyperplasia, pre-neoplasia foci formation, cancer cell migration, neo-angiogenesis, immune cell chemoattraction and the establishment of an immunosuppressive environment and initiation of a pre-metastatic niche. In one decade, RANK/RANKL has not only transformed our vision of bone biology but has also strengthened the notion of “seed and soil”, conventionally used to explain the metastatic process. Targeting RANK/RANKL signalling has already shown its therapeutic efficacy in osteoporotic patients and its clinical advantages in the management of bone metastases from breast and prostate carcinomas. Current ongoing clinical trials will be crucial for better defining its potential side effects after long termuse.

## Abbreviations

EMT: epithelial mesenchymal transition
LRG4: leucine-rich repeat-containing G-protein-coupled receptor 4
OPG: osteoprotegerin
OPGL: osteoprotegerin ligand
RANK: receptor activator of nuclear factor-κB
RANKL: receptor activator nuclear factor-κB ligand
TAM: tumour-associated macrophage
TNF-α: tumour necrosis factor-α
TRAF: TNF-receptor associated factor
TRAIL: TNF related apoptosis inducing ligand
TRANCE: tumour necrosis factor-related activation-induced cytokine
